# Quality of Life in Patients with Chronic Psychotic Disorders: A Practical Model for Interventions in Romanian Mental Health Centers

**DOI:** 10.3390/medicina58050615

**Published:** 2022-04-28

**Authors:** Marinela Hurmuz, Mirela Frandes, Anca-Livia Panfil, Ileana-Pepita Stoica, Cristina Bredicean, Catalina Giurgi-Oncu, Ion Papava, Aurel Nirestean

**Affiliations:** 1 Discipline of Psychiatry, Department of Clinical Sciences, “George Emil Palade” University of Medicine, Pharmacy, Science and Technology of Targu Mures, 38 Str. G. Marinescu, 540139 Targu Mures, Romania; marinelahurmuz@gmail.com (M.H.); aurel.nirestean@umfst.ro (A.N.); 2 Mental Health Center No. 1, “Pius Branzeu” Timis County Emergency Clinical Hospital, 156 Bd. Liviu Rebreanu, 300182 Timisoara, Romania; pepita17stoica@yahoo.com; 3 Department of Functional Sciences—Medical Informatics and Biostatistics, “Victor Babes” University of Medicine and Pharmacy, 2 Sq. Eftimie Murgu, 300041 Timisoara, Romania; 4 Liaison Psychiatry Department, “Pius Branzeu” Timis County Emergency Clinical Hospital, 156 Bd. Liviu Rebreanu, 300182 Timisoara, Romania; anca.livia.panfil@gmail.com; 5 Department of Neuroscience, NEUROPSY-COG Center for Cognitive Research in Neuropsychiatric Pathology, “Victor Babes” University of Medicine and Pharmacy, 2 Sq. Eftimie Murgu, 300041 Timisoara, Romania; bredicean.ana@umft.ro (C.B.); catalina.giurgi@umft.ro (C.G.-O.); papava.ion@umft.ro (I.P.); 6 “Dr. Victor Popescu” Emergency Military Clinical Hospital, 7 Str. G. Lazăr, 300080 Timisoara, Romania; 7 “Eduard Pamfil” Psychiatric Clinic, “Pius Branzeu” Timis County Emergency Clinical Hospital, 156 Bd. Liviu Rebreanu, 300182 Timisoara, Romania

**Keywords:** quality of life, psychosis, recovery, social skills, conceptual skills, systemic support

## Abstract

*Background and Objectives*: Current psychiatric care is increasingly focusing on patients’ quality of life (QoL). Research is still trying to determine the main factors which influence QoL. The present study aims to assess the QoL of patients with chronic psychotic-spectrum disorders, as well as its relation to symptomatology, functionality, adaptive behavior, and perceived level of recovery. *Materials and Methods*: The study included a sample of 78 patients with chronic psychosis. Symptomatology and illness severity were assessed with the Positive and Negative Syndrome Scale (PANSS) and the Clinical Global Impression Scale-Severity (CGI-S) scales, respectively. The Global Assessment of Functioning Scale (GAFS) and the Adaptive Behavior Assessment System II (ABAS-II)-Adult Form were used for the assessment of patients’ functionality, and the Quality-of-Life Inventory (QOLI) scale was applied for the evaluation of QoL. *Results*: According to the CGI-Severity scale, 20.5% of the patients were borderline mentally ill, and 24.4% were mildly ill. The highest number of patients (34.6%) were moderately ill, while 14.1% and 2.6% were markedly ill and severely ill, respectively. Among the moderately ill patients, more than half (63%) were patients with schizophrenia, 18.5% were patients with delusional disorder, and 18.5% were patients with schizoaffective disorder. Most of the patients (43.6%) presented moderate functioning deficiency, while 38.5% of the patients presented severe deficiency, according to the GAFS score. When assessed with the ABAS, we observed that almost half of the patients (44.9%) showed an average functioning across skill areas in the conceptual, social, and practical domains. A percent of 67.9% of the patients presented an average QoL, while 15.4% and 12.8% showed a very low and low QoL. QoL was not influenced by the patients’ symptomatology, gender, and education level. Having children, family support, better social and conceptual skills, and a higher perceived level of recovery was correlated with an increased QoL, hierarchical multiple regression R^2^ = 0.379, *F*(9, 68) = 2.616, and *p* = 0.012. *Conclusions*: Psychiatric interventions in psychosis should focus not only on symptoms’ control, but also on improving social and family support, as well as adaptive skills to increase the patients’ QoL.

## 1. Introduction

The interventions in psychosis have continuously changed over time. The remission of symptoms is no longer the main and only target. Current care focuses more on improving patients’ functionality, quality of life, and recovery [1]. Quality of life (QoL) is challenging to define and constantly reconsidered. It is known to have a broad meaning and to include many aspects of life. Discussions around subjective and objective QoL, the different domains of QoL, the cultural influences, and the heterogeneity of the assessment instruments have been carried out [2,3,4]. World Health Organization defines QoL as "individuals’ perceptions of their position in life in the context of the culture and value systems in which they live and in relation to their goals, expectations, standards, and concerns”. It is a broad ranging concept incorporating in a complex way the person’s physical health, psychological state, level of independence, social relationships, personal beliefs, and his or her relationship to salient features of the environment [5].

Psychotic-spectrum disorders are severe psychiatric disorders that can impair the person’s life on different levels, e.g., personal, familial, professional, social, and economic [6,7]. All the changes caused by the disorder imply a continuous process of reorganization and adjustment that needs to be supported by specific interventions, to maintain and/or improve patients’ QoL.

Many debates are ongoing regarding the impact psychiatric symptoms might have on how a person evaluates his or her life and well-being, the factors influencing the QoL of persons with psychosis, or the best interventions to improve one’s QoL [8,9]. Symptomatology is one significant variable to be studied concerning QoL in psychiatric disorders. However, results in this area are contradictory. Studies found that positive symptoms have a more significant impact on QoL [10,11,12,13], while others showed that negative, cognitive, or depressive symptoms were more critical [14,15]. Other studies did not correlate between different symptoms and QoL [16,17]. The heterogeneity of results might be due to differences in methodology and the co-influence of other variables [18,19]. Although it is probably the most extensively studied factor related to QoL, symptomatology alone is insufficient to explain the variance in these patients’ QoL. Other clinical, socio-demographic, and psycho-social factors were found to be relevant.

The duration of the disorder, severity of illness, long-term hospitalizations, and adverse reactions to treatment have all been negatively correlated with QoL [20,21,22]. The development of new medications and interventions led to better clinical responses and functionality, and, subsequently, improving QoL. Similarly, socio-demographic factors, including male gender, unemployment, and stigma have been reported to lead to poorer QoL [23,24]. Factors consistently shown to be associated with higher QoL were family and social support, employment, being in a relationship, and a good social functioning [25,26,27,28].

The issue of the validity and reliability of self-assessment tools for evaluation of both functioning level and QoL in patients with psychotic disorders has been much debated. Some authors doubt the use of self-reported measures and suggest that patients might under-estimate or over-estimate their functioning level and QoL, depending on clinical factors, such as insight, cognitive deficits, or severity of symptoms [29,30,31]. Others reported a good concordance between self-evaluation of functioning and informant reports in clinically stable outpatients [32] or concluded that patients can accurately rate their QoL [33].

The development of Mental Health Centers is viewed as a primary step in the deinstitutionalization process, providing rehabilitation and community-oriented services in Romania. Psychiatric interventions in both inpatient and outpatient settings are still mainly focused on clinical recovery. Mental Health Centers are departments of General County Hospitals that provide psychiatric, psychological, social, and rehabilitation services. Patients’ education, accessibility, and addressability to this type of service are still insufficient, making it difficult, but even more important to assess and address their QoL. Patients’ QoL is considered an important variable to evaluate the efficacy and quality of mental health services [34]. The development of proper interventions that target the improvement of QoL implies a thorough assessment of the needs and resources that exist in different settings. Research focusing on QoL in Romanian patients with psychotic spectrum disorders is scarce [35]. To our knowledge, this is the first study that both assesses QoL, in relation to a variety of factors, and highlights the possible intervention targets for a higher QoL in patients with this pathology. Thus, it supports the efforts towards improving community mental health care in our country, Mental Health Centers having a central role in this process.

The present study aimed to assess QoL and its relation to socio-demographic factors, functionality, adaptive behavior, and perceived level of recovery for a sample of patients with psychotic-spectrum disorders monitored in the Mental Health Centre in Timisoara, Romania. Secondly, we aimed to develop a hierarchical model to highlight the focus points needed to improve the patients’ QoL.

## 2. Materials and Methods

### 2.1. Study Design and Sample

The study evaluated 78 patients (46 males and 32 females) monitored by the Mental Health Centre No. 1 in Timisoara, Romania. The inclusion criteria used were: age between 18 and 65; a diagnosis of schizophrenia, schizoaffective disorder, or delusional disorder according to the ICD-10 criteria [36]; the absence of an acute psychotic episode at the moment of evaluation (patients with persistent psychotic symptoms were included); the absence of physical comorbidities known to have the potential of altering the patient’s mental state (e.g., epilepsy, Parkinson’s disease, degenerative neurological diseases, systemic autoimmune diseases, disorders of the thyroid and parathyroid glands); and the absence of the following comorbid psychiatric disorders: organic mental disorders, cognitive and behavioral disorders due to psychoactive substance use and/or intellectual developmental disorders. We assessed patients who met the criteria and consented to participate. All data were anonymized before analysis. No sensitive participant information is included in the article. All participants signed written informed consents before inclusion in the study. The study was conducted in accordance with the Declaration of Helsinki and approved by the Ethics Committee of the Timis County Emergency Clinical Hospital.

### 2.2. Assessment of Symptomatology

Current symptomatology was assessed with the Positive and Negative Syndrome Scale (PANSS) [37], which has 30 items rated on a Likert-like severity scale from 1 (absent) to 7 (severe), structured in 3 sub-scales evaluating: the positive symptoms (7 items e.g., delusions, conceptual disorganization, and suspiciousness/persecution), the negative symptoms (7 items, e.g., emotional withdrawal, poor rapport, and difficulty in abstract thinking) and the general psychopathology symptoms (16 items, e.g., anxiety, depression, poor attention, and disturbance of volition). The PANSS scale is one of the most widely used scales for the assessment of psychopathology in schizophrenia and other psychotic-spectrum disorders, its cross-cultural validity being proven by evidence-based research [38].

The Clinical Global Impression Scale–Severity (CGI-S) [39] was used to assess the illness severity on a 7-point Linkert-like scale, using a range of responses from 1 (normal) to 7 (severely ill). The CGI-S has demonstrated good psychometric properties and correlations with the PANSS [40], having proven its utility in clinical practice and research. Both instruments are hetero-assessment tools. The psychiatrist who evaluated the clinical state of the patients used the validated Romanian translations of these scales.

### 2.3. Assessment of Functionality

The Global Assessment of Functioning Scale (GAFS) [41] and the Adaptive Behavior Assessment System II (ABAS-II)–Adult Form [42] were used to assess the patients’ functionality. The GAFS is one of the most frequently used scales to rate the person’s occupational, psychological, and social functioning, with scores ranging from 100 (extremely high functioning) to 1 (severely impaired). This is a hetero-assessment tool that is part of the DSM IV-TR manual. The Romanian version of the GAFS was used in this study. ABAS II is a multifunctional instrument that can evaluate daily living skills. It covers 3 broad domains and 10 skill areas within these domains: conceptual (communication, functional academics, and self-direction), social (social, and leisure), and practical (self-care, home/school living, community use, work, health, and safety). The ABAS-II assesses persons with multiple disorders, including mental health ones. It can be completed by the patient (if capable) or by a household member. In this study, the questionnaire was completed by the patient. The raw scores were converted to standard scores and then composite score profiles. A total score (the General Adaptive Composite), test-age equivalents for skill area scores, age-based percentile ranks for the domain, and composite scores are provided. All scores can be categorized descriptively (Extremely Low, Borderline, Below Average, Average, Above Average, Superior, or Very Superior). The test also allows planning and monitoring interventions. The instrument was translated and validated for the Romanian population [43].

### 2.4. Assessment of Quality of Life

The Quality-of-Life Inventory (QOLI) [44] was used to evaluate the patients’ quality of life. It assesses well-being and satisfaction with life in 16 domains, including health, self-esteem, goals and values, money, work, play, learning, creativity, helping, love, friends, children, relatives, home, neighborhood, and community. The respondent is asked to rate the importance of each domain for their happiness (on a 3-point rating scale) and the level of satisfaction with each domain (on a 6-point rating scale). Importance scores are multiplied by satisfaction scores for each domain, obtaining a weighted average score. The overall raw score represents the mean of the weighted average scores, excluding the areas rated as unimportant by the patient. The raw score is converted to a T score, according to standardized corresponding values provided in the QOLI Manual and adapted for the Romanian population. The T score is then categorized as Very Low, Low, Average, High, and Very High. At the end of the questionnaire, the patients are also encouraged to describe the specific problems they encounter in each domain. QOLI can further be used to specifically address these problems. The QOLI was translated and validated for the Romanian population [45].

Each patient was also asked to assess their level of recovery in the moment of evaluation, based on their experience with the disorder, on a visual analog scale ranging from 0% (not at all recovered) to 100% (completely recovered). The scale was designed by the authors of the study.

### 2.5. Statistical Analysis

The collected data were described according to their statistical type, namely, continuous variables with Gaussian distribution were presented as mean (standard deviation), while the variables without Gaussian distribution were presented as median (interquartile range). The categorical variables were presented as frequency (percentage). The normality of data was tested using the Kolmogorov–Smirnov’s, and the equality of variances was assessed by using Levene’s test.

The patients with psychotic-spectrum disorders were statistically compared for significant differences by applying the student’s *t*-test (two means, Gaussian populations), Mann–Whitney U test (two medians, non-Gaussian populations), and Pearson chi-square or Fisher’s exact test (proportions). Comparison between patients with schizophrenia, patients with delusional disorder, and patients with schizoaffective disorder was done by applying the independent samples Kruskal–Wallis H test. Correlations of QoL with socio demo graphic, clinical, and functioning characteristics were assessed by computing the Spearman’s rho coefficient.

A hierarchical multiple regression was conducted to determine the factors influencing the prediction of the overall life satisfaction. The linearity was assessed by inspecting the partial regression plots and a plot of studentized residuals against the predicted values. The independence of residuals was assessed by computing the Durbin–Watson statistic. The homoscedasticity was assessed by visual inspection of a plot of studentized residuals versus unstandardized predicted values. The multicollinearity was assessed by evaluating the tolerance values. The normality was tested by inspecting the Q-Q Plot.

Data were analyzed using the SPSS v.17 software (SPSS Inc., Chicago, IL, USA). A *p*-value < 0.05 was considered the threshold for statistical significance, and a confidence level of 0.95 was considered for estimating intervals.

## 3. Results

### 3.1. Sociodemographic Characteristics

The sample included 78 patients with psychotic-spectrum disorders, aged 22 to 65 years, mean of 48.91 (±10.47) years, 95% CI (46.55; 51.27). More than half of the patients (59%) were males. Most of the patients (70.5%) were single, divorced, or widowed, and only 10.3% were employed. A high percentage of the patients (88.5%) reported having family support (Table 1).

The median age of illness onset was 27 (21.00–33.00) years, and the median number of the evolution of the disorder was 20 (14.00–28.00) years (Table 2). 59% of the patients had a diagnosis of schizophrenia.

According to the CGI-Severity scale, 20.5% of the patients were borderline mentally ill, and 24.4% were mildly ill. The highest number of patients (34.6%) were moderately ill, while 14.1% and 2.6% were markedly ill and severely ill, respectively. Among the moderately ill patients, more than half (63%) were patients with schizophrenia, 18.5% were patients with delusional disorder, and 18.5% were patients with schizoaffective disorder. All patients severely ill were patients with schizophrenia.

Most of the patients (48.7%) had minimal positive symptoms, 29.5% had mild positive symptoms, and only 5.1% had moderate–severe or severe–extreme symptoms. Regarding negative symptoms, 57.7% of the patients had minimal or mild symptoms, 26,9% presented moderate symptoms, and 15.4% showed moderate–severe or severe–extreme ones.

Most of the patients (43.6%) presented moderate functioning deficiency, while 38.5% of the patients presented severe deficiency, according to the GAFS score. When assessed with the ABAS, we observed that almost half of the patients (44.9%) showed an average functioning across skill areas in the conceptual, social, and practical domains and a general adaptive composite that includes all the skills areas (Table 3).

When comparing the functioning capacity measured by GAFS score between patients with schizophrenia, delusional disorder, and schizoaffective disorder, we observed no significant differences (Kruskal–Wallis H test, H(2) = 1.874, *p* = 0.392). Also, distributions of functioning capacity were similar for all groups when assessed by ABAS-GAC score (Kruskal–Wallis H test, H(2) = 0.803, *p* = 0.669).There were no significant differences between groups when considering the conceptual adaptive domains (Kruskal–Wallis H test, H(2) = 1.732, *p* = 0.421), the social domains (Kruskal–Wallis H test, H(2) = 1.708, *p* = 0.426), and the practical domains (Kruskal–Wallis H test, H(2) = 0.161, *p* = 0.923).

Most of the patients (67.9%) presented an average overall QoL (T score = 43–57), while 15.4% and 12.8% showed a very low and low overall QoL. We found no significant differences between QoL components of patients with different psychotic-spectrum disorders (Table 4).

There were no significant differences between the QoL of male patients compared with female patients, mean rank 38.84 vs. 40.45 (Mann–Whitney U test, U = 766.5, z = 0.310, *p* = 0.756). No significant differences were found between the QoL of patients living in urban areas compared with those living in a rural area, mean rank 40.06 vs. 37.5 (Mann–Whitney U test, U = 484.5, z = −0.412, *p* = 0.680). On the contrary, we observed that patients with children presented significantly higher QoL than patients without children, mean rank 49.07 vs. 33.52 (Mann–Whitney U test, U = 1,007, z = 2.952, *p* = 0.003).

Patients without a life partner (single, widowed, or divorced) presented significantly lower scores of QoL than patients who were married or in a cohabiting partnership, 47.16 vs. 51.83 (Mann–Whitney U test, *p* = 0.012). Patients who were employed presented slightly higher scores of QoL than patients without a job or retired, 52.11 vs. 48.07 (Mann–Whitney U test, *p* = 0.112).

There were no significant differences between the QoL of patients with different illness severity scores measured by CGI-S, Kruskal–Wallis H test, H(5) = 6.818, *p* = 0.235. Distributions of QoL were not significantly different across different categories of functioning capacity, mean rank GSFS score, 51.75 vs. 45.45 vs. 34.31 vs. 37.29, Kruskal–Wallis H test, H(3) = 4.565, *p* = 0.207.

### 3.2. Correlations of QoL with Sociodemographic, Clinical, and Functioning Characteristics

QoL was negatively associated with illness severity, but without significance, when considering all the patients with psychotic-spectrum disorders (Spearman’s rho = −0.212, *p* = 0.062). The education level was poorly negatively correlated with the level of QoL without being significant (Spearman’s rho = −0.133, *p* = 0.247).

Regarding symptoms, we observed no significant association between the QoL and positive symptoms (Spearman’s rho = −0.201, *p* = 0.078) or negative symptoms (Spearman’s rho = −0.160, *p* = 0.161). The PANSS-G scores were weakly negatively correlated with QoL (Spearman’s rho = −0.168, *p* = 0.142).

QoL was positively associated with functioning capacity (Spearman’s rho = 0.281, *p* = 0.013). Also, it was positively significantly associated with the general adaptive skills (Spearman’s rho = 0.292, *p* = 0.009), with the adaptive abilities in conceptual domains (Spearman’s rho = 0.286, *p* = 0.011) and the adaptive skills in social domains (Spearman’s rho = 0.360, *p* = 0.001). We found a positive association between QoL and adaptive skills in functional domains, but without significance (Spearman’s rho = 0.175, *p* = 0.126).

The degree of recovery perceived by the patient was positively and significantly correlated with QoL (Spearman’s rho = 0.262, *p* = 0.020).

### 3.3. Factors Influencing QoL

We applied hierarchical multiple regression to determine if the addition of positive and negative symptoms, illness severity, disease duration, functioning capacity, and children and family support improved the prediction of QoL over and above adaptive skills alone. Table 5 presents the details of the full regression model.

The presence of linearity was assessed by inspecting the partial regression plots and a plot of studentized residuals against the predicted values was observed. At the same time, the residuals were independent, as assessed by a Durbin–Watson statistic of 2.031. In addition, we observed homoscedasticity, as evaluated by visual inspection of a plot of studentized residuals versus unstandardized predicted values. There were three studentized deleted residuals greater than ±2 SD.

The complete model including adaptive skills, functioning capacity, positive and negative symptoms, illness severity, disease duration, having children, family support, and social support to predict QoL was statistically significant, R^2^ = 0.257, F(9, 68) = 2.616, *p* = 0.012; adjusted R^2^ = 0.159.

The addition of illness severity and positive and negative symptoms to the prediction led to an insignificant change in R^2^. The addition of family support and children to the prediction led to a statistically significant increase in R^2^ of 0.122, F(2, 68) = 5.605, *p* = 0.006. Regression coefficients and standard errors are listed in Table 5.

We observed that an increase in one SD’s conceptual adaptive skills and adaptive social skills was significantly associated with an increase QoL of 0.010 SD and 0.135 SD, respectively (Table 5). At the same time, an increase in the functioning capacity of one SD was associated with an increase in QoL of 0.067 SD. In addition, we observed that an increase in illness severity of one SD was associated with a decrease in QoL of 1.448 SD. An increase of illness duration of one SD was associated with a decrease of QoL of 0.034 SD. Moreover, we observed that having children significantly increased QoL of 5.653 SD.

## 4. Discussion

The study aimed to assess QoL and its relation to different factors, such as symptomatology, level of functionality and adaptive behaviors, and perceived level of recovery in a sample of patients with psychotic-spectrum disorders monitored in the Mental Health Centre in Timisoara, Romania. In addition, it aimed to develop a hierarchical model to highlight the main points needed to improve the patients’ QoL.

Almost 68% of the subjects in our study rated their QoL on an average level, and 3.8% on a high level as compared with the data provided for the Romanian general population. This is an interesting finding, as research results vary in this matter. The most common belief, supported by different studies, is that persons with schizophrenia and other psychotic disorders usually have a lower QoL than the general population, due to the negative impact of the disorder on people’s lives in all areas of living [18]. Still, others reported that these patients rate their QoL on an average or even higher level than the general population, especially patients living in the community [46]. Our results can be explained from different perspectives. The patients in our study were clinically stable and frequent users of the Mental Health Center’s services, including psychological and social services that support their well-being. In addition, during the evolution of the disorder, patients can also develop adaptive mechanisms skills and resilience, besides its negative impact on their lives. QoL scores were not significantly different according to the patients’ diagnoses, though these results might be influenced by the small number of subjects with each diagnostic.

We found no correlation between gender and QoL, nor between the location of living (rural or urban) and QoL. A percent of 75.7% of the patients were retired due to their psychiatric disorder, and only 29.5% were married or in a relationship. These facts are consistent with other research data showing that psychotic disorders harm the patients’ professional functioning and personal life [25,47,48,49]. Symptoms, impairment of social-functioning skills, stigma, insufficient rehabilitation services, and poor social support might contribute to this situation. In contrast, subjects who were married or had an intimate relationship, who had children and were employed reported significantly higher QoL scores. These results are supported by other studies that proved the importance of relationships for one’s QoL [50,51]. Being married or having a partner was correlated with higher QoL and fewer psychotic experiences. [51,52]. This is understandable, as significant relationships can offer emotional support, sustain, and help improve patients’ functioning and increase motivation for recovery. In addition, being employed can help patients maintain social relationships, feel useful and develop their cognitive and social skills. Although being a parent with mental illness may lead to even more challenges and difficulties that can negatively impact QoL, it can also promote one’s adaptive skills, sense of efficacy and confidence, and offer meaning and purpose [53,54]. Thus, it might contribute to a higher QoL. These results also highlight the importance of vocational rehabilitation programs and of interventions that support the whole family system.

Regarding education, we found a negative correlation between level of education and QoL, even though it was not statistically significant, most probably due to the small sample. While some studies reported similar results [23], others found that a higher level of education is connected to a better QoL [55]. It might be that a higher education level leads to higher expectations and social demands. Moreover, it can be connected to a better insight and knowledge regarding the impact of the disorder on one’s life. Thus, these patients might report a lower QoL.

From a clinical point of view, most of the patients had minimal or mild positive and negative symptoms. According to the CGI score, patients with schizophrenia were more severely ill than those with delusional or schizoaffective disorder. In our study, symptoms and clinical global severity of the illness have not significantly influenced the QoL. However, a deterioration in the clinical state was associated with a decrease in QoL. This finding is in accordance with other study results that reported a negative correlation between symptoms’ severity and QoL [26]. Regarding the influence of symptoms on QoL, scientific data are heterogenous, probably due to differences in methodology and definitions of QoL. Some studies showed that positive psychotic symptoms led to a reduction in QoL [10,11,12]; others reported no influence [13,14,27]. Regarding negative symptoms, results were also contradictory, correlated to a poorer QoL [14,16,56] or, according to other findings [14], not having a significant impact on QoL. Other studies also considered depressive and cognitive symptoms, finding substantial correlations [13,15]. In our study, the correlation between the general symptomatology (PANSS-G score) and QoL was weak, while other studies found a strong negative association [57]. Results might differ, when depression, anxiety and cognitive symptoms are assessed using specific evaluation instruments, these aspects being relevant issues to consider in future research. Some studies also made a difference between the influence of symptoms on subjective or objective QoL, stating that depressive symptoms have more impact on subjective QoL, and negative symptoms affect more the objective QoL [4]. In our study, only 5.1% of the subjects had moderate–severe or severe–extreme positive symptoms, and only 15.4% presented moderate–severe or severe–extreme negative symptoms, which might contribute to the absence of a significant correlation between symptoms and QoL, as only an increase in illness severity would lead to a decrease in QoL. The clinical state of the patients has, without a doubt, great importance for their well-being and makes an important target for treatment. Still, our study comes in the same line with other findings, highlighting that symptom control is insufficient when therapeutic interventions focus on the patient’s overall well-being [58,59].

Psychotic disorders can impact the person’s global functionality [60]. Our study also showed that 81.1% of the patients presented a moderate or severe functional deficiency when assessed by the psychiatrist with the GAF scale. When patients assessed their functionality with the ABAS, we observed an average functionality across skills areas in all domains for almost half of them, with 25% scoring for high and above-average functionality and 29.5% scoring for a below-average, low, or extremely low functionality. The differences between the instruments used to measure the level of functioning, which can lead to the variance of results, were also underlined in other studies [61]. The GAFS also includes symptomatology in the assessment of functioning level, which might influence the scores given by the clinician. Some research findings also suggested that patients, especially those with schizophrenia, might over-estimate their functioning level [29]. The mediating effect of symptomatology, particularly negative, cognitive symptoms, and poor insight could explain this fact [30,61]. The ABAS II instrument includes many items related to basic skills in daily living activities, academics, self-care, or communication, which are usually affected in more severe cases of psychotic disorders. Our study only included a few patients with severe symptoms, with a median of 12 years of education, and an adequate family support for most of them. These facts might contribute to a higher level of functioning in the previously mentioned areas. The lowest scores were observed in the social domain. These results are in line with other research, showing the important impact of psychotic-spectrum disorders on patients’ relationships and social skills [62]. When correlated with the QoL, the study also reported differences between the assessment tools. While the GAFS scores did not influence QoL, QoL scores were significantly correlated with the ABAS domains (the overall adaptive skills, the adaptive skills in conceptual and social domains, but not in the practical domains). An increase in the functioning capacity, especially in the conceptual and social adaptive skills, was reported to increase QoL. There are many contradictory results in the literature concerning the relationship between functionality and QoL.

Some studies support our findings in reporting positive correlations between functioning level and QoL, especially when considering the social domain [13,15,58,60,63]. In contrast, others reported no correlation, when controlling for symptomatic level [4,27]. The conceptual area in the ABAS II report includes communication, functional academics, and self-direction domains. The result indicating that better skills in these areas would lead to higher QoL can be explained by the fact that they are connected to social relationships, self-control skills, independent living, cognitive skills, and involvement in different activities and projects, which might also be mediating factors for QoL.

Our results also showed a significantly positive correlation between the patients’ perceived level of recovery and QoL. This finding supports the idea of personal recovery and of the importance of the patient’s perception regarding their personal recovery, besides the objective evaluation of clinical and functional recovery. It also emphasizes the value of recovery-oriented therapeutic approaches.

The hierarchical regression model highlighted the importance of considering multiple factors, when focusing on the patient’s QoL. The complete model included adaptive skills, functioning capacity, positive and negative symptoms, illness severity, illness duration, having children, family support, and social support, and it led to a significantly prediction of QoL. According to our model, factors related to functionality (global functioning capacity, and adaptive skills in social and conceptual areas) and significant relationships (having children, perceived family, and social support) have a greater importance than clinical factors for the improvement of QoL. This is an important aspect, as in our country treatment still focuses mostly on symptom control. Results might differ for persons with more severe symptoms, in the acute phases of illness or for inpatient settings, where symptoms’ reduction is essential as a primary intervention. Moreover, our model also showed that an increase in illness severity and duration would negatively impact QoL, underlining the importance of early intervention. Still, when focusing on persons from outpatient settings, such as Mental Health Centers, interventions need to go beyond the clinical aspects, and approach the disorder at a systemic level. Our results are in accordance with other studies that proposed multidimensional models of QoL and the importance of the functioning level for QoL. In a recent study [64] that proposed a model including both objective (e.g., medication used, negative symptoms, course of disorder, and physical comorbidities) and subjective components (e.g., perception of loneliness) that might impact QoL, global functioning has been found to play a mediating role. Another study [15] concluded that psychotic and cognitive symptoms only had a weak influence on QoL, which was also mediated by impaired functioning.

Developing mental health programs that include patients’ families in the therapeutic plan and that focus on social-skills training could have a great impact on improving patients’ QoL. Beyond other important factors that contribute to the patients’ QoL, it is ultimately the presence and quality of relationships that might connect all these factors.

There are certain limitations of our study. One of them is the small sample size. Therefore, the results cannot be generalized for all categories of patients with psychotic-spectrum disorders, although the sample might be representative for patients monitored in Mental Health Centers who have a chronic psychotic disorder and a relatively stable clinical state. Our findings provide valuable information that can support the development of recovery-oriented interventions. Another limitation refers to the assessment scales used in the study. The visual analog scale used for the recovery assessment was not validated. Although research showed that visual analog scales are valid and reliable instruments to assess subjective characteristics or attitudes [65], a more detailed approach is necessary for a deeper understanding of the relationship between QoL and recovery in this category of individuals. The ABAS II and QOLI instruments were not particularly validated for persons with psychotic disorders, but they were both translated and validated for the Romanian population. Level of functioning, QoL and standards of living can vary in different countries due to socio–economic factors. Therefore, the validation of the assessment tools in these domains are important aspects to consider for the quality of research.

## 5. Conclusions

QoL is influenced by socio–demographic, clinical, and functioning-related factors, such as professional and marital status, functioning level, adaptive skills in different areas of life, and the degree of perceived recovery. Symptoms’ control in psychotic disorders proves to be insufficient for assessing one’s QoL, at least in patients who are not severely ill. Having children, family and social support, a good functioning level, and adaptive skills in both personal and professional domains are essential factors, contributing to an increased QoL.

Improving the QoL of patients with psychotic disorders is a necessary target to consider when developing a therapeutic plan. The assessment of factors that impact QoL from a multidimensional perspective can offer valuable information for developing specific interventions. A recovery-oriented approach that pertains to several domains, such as social skills training, involving the family in the therapeutic plan, and promoting systemic support is needed in Romania, where significant systemic changes are still in process.

## Figures and Tables

**Table 1:** Sociodemographic characteristics of the patients.

Number of Participants	*N* = 78
Age [years] ^(a)^	48.91 (±10.47)
Gender (male) ^(c)^	46 (59%)
Location (urban) ^(c)^	61 (78.2%)
Education [years] ^(b)^	12 (11–13)
Civil status ^(c)^	
Married	15 (19.2%)
Cohabiting partnership	8 (10.3%)
Single	35 (44.9%)
Widow	4 (5.1%)
Divorced	16 (20.5%)
Children ^(c)^	30 (38.5%)
Professional status ^(c)^	
Employed/Self-employed	8 (10.3%)
Unemployed/Retired	70 (89.74%)
Family support ^(c)^	69 (88.5%)
Social support ^(c)^	45 (57.7%)

^(a)^ Continuous variables (with Gaussian distribution) are indicated by their mean (standard deviation). ^(b)^ Continuous variables (with non-Gaussian distribution) are indicated by their median (interquartile range-IQR). ^(c)^ Categorical variables are presented by absolute frequency and percentage in the sample.

**Table 2:** Clinical characteristics of the sample.

Parameters	*N* = 78
Diagnostic ^(b)^	
Schizophrenia	46 (59%)
Delusional disorder	16 (20.5%)
Schizoaffective Disorder	16 (20.5%)
Age at onset ^(a)^	27 (21–33)
Duration of disorder (years) ^(a)^	20 (14–28)
Illness severity (CGI-S score) ^(a)^	4 (3–4)
PANSS-Positive score ^(a)^	14 (10–20)
PANSS-Negative score ^(a)^	19 (14–24)
PANSS-General psychopathology score ^(a)^	36 (32–44)
PANSS-total score ^(a)^	70 (59–84)
Recovery score ^(a)^	70 (50–80)

Observations: ^(a)^ Continuous variables (with non-Gaussian distribution) are indicated by their median (interquartile range-IQR). ^(b)^ Categorical variables are presented by absolute frequency and percentage in the sample. CGI-S score (ranges 1–7): 1 = normal, not at all ill; 2 = borderline mentally ill; 3 = mildly ill; 4 = moderately ill; 5 = markedly ill; 6 = severely ill; and 7 = among the most extremely ill patients. PANSS-Positive scale (ranges 7–49): 7–13 absent, 14–20 minimal, 21–27 mild, 28–34 moderate, 35–41 moderate-severe, and 42–49 severe-extreme. PANSS-Negative (ranges 7–49): 7–13 absent, 14–20 minimal, 21–27 mild, 28–34 moderate, 35–41 moderate-severe, and 42–49 severe-extreme. PANSS-General psychopathology scale (ranges 16–112). Abbreviations: CGI-S—Clinical Global Impression-Severity scale; and PANSS-positive and negative syndrome scale.

**Table 3:** Functioning characteristics of the patients with psychotic-spectrum disorders.

Parameters ^(a)^	Total (*N* = 78)	Schizophrenia (*N* = 46)	Delusional Disorder (*N* = 16)	Schizoaffective Disorder (*N* = 16)	*p*-Value ^(b)^
GAFS score	60 (50–70)	60 (45–70)	60 (55–72.5)	60 (60–67.5)	0.392
ABAS-GAC	93 (82–106)	93 (82–104)	93 (79–105.5)	98 (83–108.5)	0.669
ABAS-Conceptual	94 (81–108)	91 (83–109)	93 (65.5–103)	100 (74.5–111.5)	0.421
ABAS-Social	83 (54–96)	83 (51–96)	84.5 (52.5–94.5)	91 (76.5–100.5)	0.426
ABAS-Practical	104 (94–111)	103 (94–112)	103 (96–110)	106 (85–111)	0.923

Observations: ^(a)^ Continuous variables (with non-Gaussian distribution) are indicated by their median (interquartile range-IQR). ^(b)^ Independent samples Kruskal–Wallis H test—comparison between patients with schizophrenia, patients with delusional disorder, and patients with schizoaffective disorder. GAFS score (ranges 1–100): 1–20 extreme deficiency, 21–40 severe deficiency, 41–60 moderate deficiency, 61–80 mild deficiency, and 81–100 without defect. Abbreviations: GAFS—a global assessment of functioning scale; ABAS-adaptive behavior assessment system; and GAC—general adaptive composite.

**Table 4:** Quality of life characteristics of the patients with psychotic-spectrum disorders.

Variables QoL ^(a)^	Total (*N* = 78)	Schizophrenia (*N* = 46)	Delusional Disorder (*N* = 16)	Schizoaffective Disorder (*N* = 16)	*p*-Value ^(b)^
Health	2 (4–4)	2 (2–4)	4 (4–4)	4 (3–5)	0.075
Self-esteem	2 (2–4)	2 (1–4)	2 (2–4)	2 (0.5–2)	0.148
Goals and values	2 (1–4)	2 (1–4)	2 (1–4)	2 (2–4)	0.878
Money	2 (0–2)	2 (0–3)	2 (0–2)	2 (0–3)	0.991
Work	2 (1–4)	2 (1–4)	2 (1–4)	2 (−1.5–5)	0.984
Play	2 (0 –4)	2 (0–3)	1.5 (0–2.5)	2.5 (0.5–4)	0.697
Learning	2 (1–4)	2 (1–4)	2 (0–3)	2 (2–4)	0.198
Creativity	2 (1–4)	2 (1–4)	2 (1–2.5)	3 (0.5–5)	0.606
Helping	2 (1–4)	2 (1–4)	2 (1.5–6)	2 (1–4)	0.682
Love	1 (−2–4)	1 (−2–2)	3 (0.5–5)	2.5 (−2–4)	0.191
Friends	2 (0–3)	1 (0–2)	2 (0–4.5)	2 (−1–4)	0.397
Children	1 (−1–4)	0 (−2–3)	6 (2–6)	0 (−2–2)	0.001
Relatives	2 (1–6)	2 (1–4)	2 (1.5–6)	2 (2–6)	0.398
Home	4 (2–6)	3 (2–6)	6 (2–6)	4 (2.5–6)	0.250
Neighborhood	2 (2–4)	2 (1–3)	2 (2–4)	3.5 (1.5–6)	0.545
Community	2 (2–4)	2 (1–3)	2 (2–6)	4 (2–5)	0.210
Total	48 (44–53)	47 (43–52)	50 (45.5–56.5)	51 (44–54.5)	0.163

Observations: ^(a)^ Variables are described by their median (interquartile range-IQR). ^(b)^ Independent samples Kruskal–Wallis H test—comparison between patients with schizophrenia, patients with delusional disorder, and patients with schizoaffective disorder. Abbreviations: QoL, quality of life.

**Table 5:** Summary of the complete hierarchical multiple regression analysis.

Variable	B	SE_B_	β
Intercept	26.212	11.503	
Adaptive skills—conceptual	0.010	0.070	−0.025
Adaptive skills—social	0.135	0.062	0.438 *
Functioning capacity	0.067	0.105	0.128
Illness severity	1.448	1.372	0.221
Positive symptoms	0.023	0.144	0.019
Negative symptoms	−0.041	0.144	−0.036
Illness duration	0.034	0.076	0.048
Family support	1.361	2.564	0.058
Children	5.653	1.721	0.366 *

Note. * *p* < 0.05; B = unstandardized regression coefficient; SEB = standard error of the coefficient; and β = standardized regression coefficient.

## Data Availability

Not applicable.
